# Aberrant cytoplasmic intron retention is a blueprint for RNA binding protein mislocalization in VCP-related amyotrophic lateral sclerosis

**DOI:** 10.1093/brain/awab078

**Published:** 2021-03-09

**Authors:** Giulia E Tyzack, Jacob Neeves, Hamish Crerar, Pierre Klein, Oliver Ziff, Doaa M Taha, Raphaëlle Luisier, Nicholas M Luscombe, Rickie Patani

**Affiliations:** 1 Human Stem Cells and Neurodegeneration Laboratory, The Francis Crick Institute, London, NW1 1AT, UK; 2 Department of Neuromuscular Diseases, Queen Square Institute of Neurology, University College London, London, WC1N 3BG, UK; 3 Zoology Department, Faculty of Science, Alexandria University, Alexandria 21511, Egypt; 4 Genomics and Health Informatics Group, Idiap Research Institute, CH - 1920 Martigny, Switzerland; 5 UCL Genetics Institute, University College London, London, WC1E 6BT, UK; 6 Okinawa Institute of Science and Technology Graduate University, Okinawa 904-0495, Japan

**Keywords:** cytoplasmic intron retention, human stem cell model, nuclear/cytoplasmic fractionation, amyotrophic lateral sclerosis, protein mislocalization

## Abstract

We recently described aberrantly increased cytoplasmic SFPQ intron-retaining transcripts (IRTs) and concurrent SFPQ protein mislocalization as new hallmarks of amyotrophic lateral sclerosis (ALS). However, the generalizability and potential roles of cytoplasmic IRTs in health and disease remain unclear. Here, using time-resolved deep sequencing of nuclear and cytoplasmic fractions of human induced pluripotent stem cells undergoing motor neurogenesis, we reveal that ALS-causing *VCP* gene mutations lead to compartment-specific aberrant accumulation of IRTs. Specifically, we identify >100 IRTs with increased cytoplasmic abundance in ALS samples. Furthermore, these aberrant cytoplasmic IRTs possess sequence-specific attributes and differential predicted binding affinity to RNA binding proteins. Remarkably, TDP-43, SFPQ and FUS—RNA binding proteins known for nuclear-to-cytoplasmic mislocalization in ALS—abundantly and specifically bind to this aberrant cytoplasmic pool of IRTs. Our data are therefore consistent with a novel role for cytoplasmic IRTs in regulating compartment-specific protein abundance. This study provides new molecular insight into potential pathomechanisms underlying ALS and highlights aberrant cytoplasmic IRTs as potential therapeutic targets.

## Teaser

Aberrant cytoplasmic SFPQ intron-retaining transcripts (IRTs) have recently been described in ALS, but their roles remained unclear. Tyzack, Neeves *et al.* now identify >100 cytoplasmic IRTs and show that they are ‘blueprints’ for ALS-related RNA binding protein mislocalization, as well as potential therapeutic targets.

## Introduction

Studies have demonstrated that intron retention is more frequent in mammals than originally recognized, affecting transcripts from a majority of genes.^1–4^ It is noteworthy that neural cells, with their exceptional polarity and compartmentalization, exhibit higher degrees of intron retention compared with other cell types.^1^^,^^2^^,^^4^ Indeed, intron retention is a prominent mode of splicing during early neuronal differentiation^2^^,^^5^ and plays a functional role in neuronal homeostasis.^1^^,^^2^^,^^4^^,^^6^ Intron retention has previously been implicated in regulating the transcriptome by coupling to RNA degradation pathways.^1^^,^^2^^,^^7–9^ Although intron-retaining transcripts (IRTs) have predominantly been identified as residing within the nucleus where they are degraded,^1–3^ there is an expanding body of evidence demonstrating the cytoplasmic localization of IRTs.^6^^,^^10–13^ However, their prevalence and role remain understudied. One of the few studies focusing on a cytoplasmic IRT showed that a retained intron in the *Calm3* transcript determined its dendritic localization,^13^ thus revealing an addressing (or ‘zip-coding’) function of cytoplasmic intron retention. This study raises the possibility of new roles for intronic RNA sequences beyond a nuclear function, and suggests that cytoplasmic intron retention programmes are relevant to human neurological function and their perturbation, therefore, to disease.

Amyotrophic lateral sclerosis (ALS) is a rapidly progressive and incurable adult-onset condition, which leads to the relatively selective degeneration of motor neurons. The molecular pathological hallmark of ALS is a nuclear-to-cytoplasmic mislocalization of key RNA binding proteins (RBPs),^5^^,^^14^^,^^15^ although the underlying mechanism for this phenomenon remains elusive. ALS-causing gene mutations implicate crucial regulators of RNA processing, which are normally expressed throughout development.^16^^,^^17^ This raises the hypothesis that post-transcriptional changes, including those occurring during neurodevelopment, may play a pivotal role in the underlying molecular pathogenesis of ALS. We recently described intron retention as the predominant splicing event characterizing early stages of motor neuron lineage restriction from human induced pluripotent stem cells (hiPSCs), which is perturbed by genetically diverse ALS-causing mutations.^5^ However, whether this process affects the nuclear or cytoplasmic subcellular compartments similarly remains unresolved. Few studies have examined compartment-specific intron retention in differentiated neurons,^6^^,^^11^ and to our knowledge, no study has comprehensively investigated cytoplasmic intron retention programmes during human motor neurogenesis, nor systematically characterized the effect of an ALS-causing mutation.

Here, we combine cellular fractionation of hiPSCs undergoing motor neurogenesis with deep RNA-sequencing (RNA-seq) of ∼100 million paired end reads per sample to gain insight into the molecular ‘logic’ governing intron retention programmes in healthy and disease states. This is a rich resource for researchers across the disciplines of basic and applied neuroscience, constituting six time points during motor neurogenesis for four control lines (from four healthy individuals) and three ALS lines (from two patients carrying mutations in the *VCP* gene), which have been fractionated into nuclear and cytoplasmic samples. Indeed, this resource allowed us to make important insights into the nature of aberrant intron retention in a human stem cell model of ALS. Specifically, we provide a taxonomy for aberrant intron retention based on nucleocytoplasmic distribution, *cis* attributes and predicted intron binding affinities to major RBPs. Remarkably, this revealed >100 IRT species in the cytoplasm of ALS cultures, suggesting that this is a more widespread phenomenon than previously recognized. Furthermore, we confirm direct binding of splicing factor proline and glutamine rich (SFPQ) protein with SFPQ IRT within the cytoplasm of our hiPSC ALS model along with preliminary evidence of a functional interaction between them. In summary, we have uncovered a novel class of cytoplasmic IRTs that exhibits predictive value for the nuclear-to-cytoplasmic mislocalization of key RBPs, a recognized molecular hallmark of ALS.

## Materials and methods

Detailed methods are provided in the Supplementary material.

### Ethics statement

Informed consent was obtained from all patients and healthy control subjects in this study. Experimental protocols were all carried out according to approved regulations and guidelines by UCLH’s National Hospital for Neurology and Neurosurgery and UCL’s Institute of Neurology joint research ethics committee (09/0272).

### Cell culture

HiPSCs were maintained on Geltrex^TM^ (Life Technologies) with Essential 8^TM^ Medium (Life Technologies), and passaged using EDTA (Life Technologies, 0.5 mM). All cell cultures were maintained at 37°C and 5% CO_2_. Directed differentiation of hiPSCs to motor neurons was performed as previously described.^18^ Details of the lines used in this study are provided in Supplementary Table 1. One of the control lines used (Control 3) is commercially available and was purchased from Thermo Fisher Scientific (cat. number A18945).

### Data availability

All sequence data for this project have been deposited at NCBI GEO database under accession number GSE152983. Additional data supporting the findings of this study are available from the corresponding authors, upon reasonable request.

## Results

### High coverage RNAseq data from nuclear and cytoplasmic fractions during human motor neurogenesis

**Figure 1:**
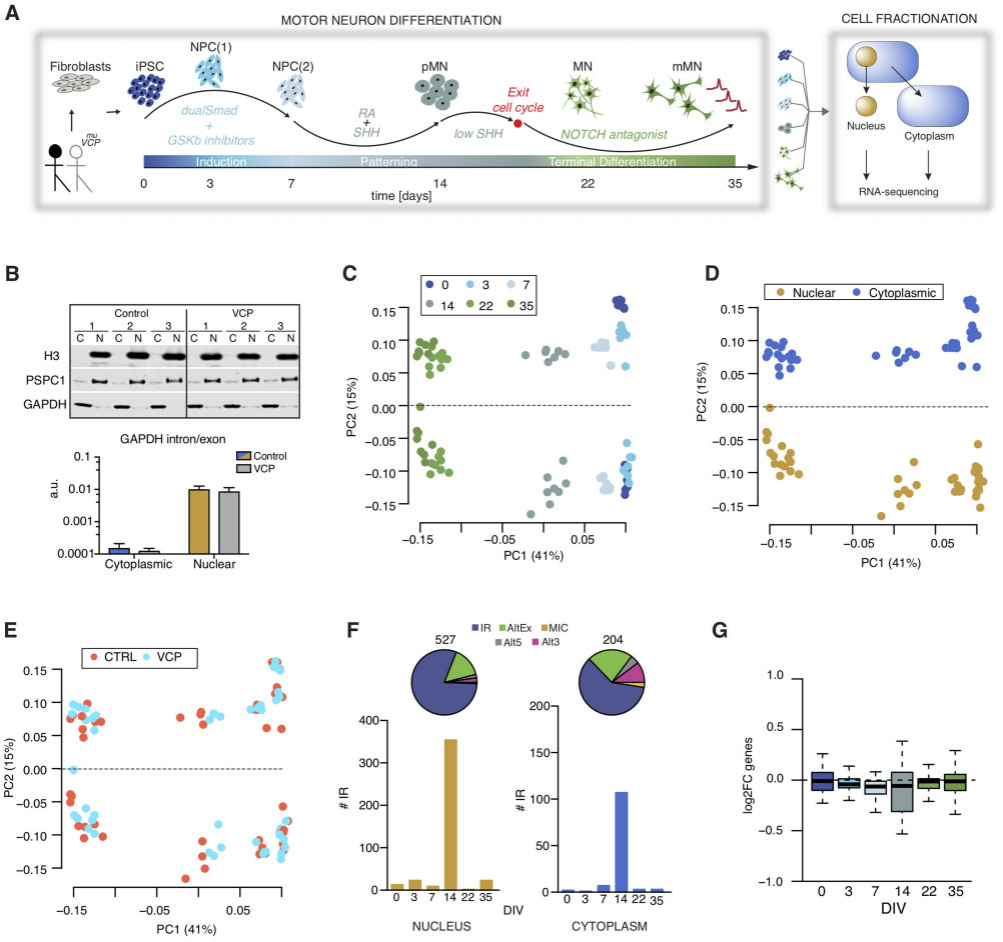
**Time-resolved cellular fractionation and RNA sequencing during human motor neurogenesis reveals widespread Aberrant cytoplasmic intron retention is a blueprint for RNA binding protein mislocalization in VCP-related ALS.** (**A**) Schematic depiction of the iPSC differentiation strategy for motor neurogenesis. Arrows indicate sampling time points in days when cells were fractionated into nuclear and cytoplasmic compartments prior to deep [poly(A)] RNA sequencing. Four iPSC clones were obtained from four different healthy controls and three iPSC clones from two ALS patients with VCP mutations: R155C and R191Q; hereafter termed VCP^*mu*^. NPCs = neural precursors; pMN = ‘patterned’ precursor motor neurons (ventral spinal cord); MN = post-mitotic but electrophysiologically inactive motor neurons; mMN = electrophysiologically active motor neurons. (**B**) Representative QC data for fractionation of samples at DIV = 14 at protein level (western blot, *top*) and RNA level [quantitative (q)PCR, *bottom*]. In the western blot, histone H3 and PSPC1 were chosen as protein markers for the nuclear faction, and GAPDH was used as a cytosolic marker. In the qPCR, the ratio between intronic and exonic GAPDH sequences was measured in both fractions to exclude the leakage of nuclear RNA into the cytosolic fraction due to disruption of nuclei during the fractionation. Data are expressed as mean ± standard deviation (SD) from four lines per group. (**C**) Singular value decomposition (SVD)performed on normalized 18 834 gene expression values across 95 samples. Samples are plotted by their coordinates along PC1 (41% of variance) and PC2 (15% of variance). Colours of data-points indicate similar time in culture: iPSC (dark blue), DIV = 3 (blue; NPC1), DIV = 7 (light blue; NPC2), DIV = 14 (grey; pMN), DIV = 22 (light green; MN) and DIV = 35 (dark green; eMN). (**D**) Same as **C** with colours of data-points indicating similar cellular fractions: nuclear fraction (gold) and cytoplasmic fraction (blue). (**E**) Same as **C** with colours of data-points indicating either control samples (red) or VCP^*mu*^ samples (blue). (**F**) *Top:* Pie charts representing proportions of included splicing events in VCP^*mu*^ at all time points of motor neurogenesis compared with age-matched control samples in nuclear (*top chart*) and cytoplasmic (*bottom chart*) fractions. Total number of events are indicated above the chart. Alt5 and Alt3 = alternative 5′ and 3′ UTR; AltEx = alternative exon; MIC = microexons; IR = intron retention. *Bottom:* Bar graphs representing the number of retained introns in VCP^*mu*^ compared to control samples at specific time points during motor neuron differentiation. Nuclear fraction (gold; *left*). Cytoplasmic fraction (blue; *right*). (**G**) Box plots showing the distributions of cytoplasmic log2 fold-changes for 72 essential splicing factor genes (Supplementary Table 9) between VCP^*mu*^ and controls.

We analysed high-throughput poly(A) RNAseq data derived from nuclear and cytoplasmic fractions of hiPSCs (Day 0), neural precursors (NPC; Days 3 and 7), ‘patterned’ precursor motor neurons (pMN; Day 14), post-mitotic but electrophysiologically immature motor neurons (MN; Day 22), and electrophysiologically active motor neurons (mMNs; Day 35). The cellular material was derived from four healthy controls and two ALS patients with *VCP* mutations: R155C and R191Q, hereafter termed VCP^*mu*^ (Fig. 1A) [95 samples from six time points and two genotypes (healthy and VCP^*mu*^-related ALS); four clones from four different healthy controls and three clones from two VCP*^mu^* patients]. Further details of the samples sequenced can be found in Supplementary Table 1 and details of the RNAseq quality control in Supplementary Table 2. Cells from each stage of differentiation were characterized as previously reported.^18^ The efficiency of cellular fractionation was assessed both at protein and RNA levels. The predominantly nuclear proteins histone H3 and PSPC1 were highly enriched in the nuclear fraction, whereas the cytosolic enzyme GAPDH was mainly detected in the cytoplasm (Fig. 1B). Similarly, the presence of *GAPDH* intronic RNA was negligible in the cytoplasm, suggesting that leakage of RNA from the nucleus to the cytoplasm due to the fractionation protocol was minimal. Importantly, the efficiency of fractionation was comparable between control and VCP*^mu^* lines. Singular value decomposition (SVD) analysis of 18 834 reliably expressed genes across the 95 samples revealed that developmental stage and cellular fraction were the largest contributors to transcriptome diversity, explaining 41% and 15% of the variance, respectively. Notably, the VCP^*mu*^ samples cluster with their age- and fraction-matched control counterparts (Fig. 1C–E). Unsupervised hierarchical clustering (Spearman rank correlation and complete-linkage clustering) of the 95 samples using 18 834 genes segregated samples by developmental stage rather than genetic background (Supplementary Fig. 1A).

### Widespread aberrant cytoplasmic intron retention in a human stem cell model of ALS

We previously identified ALS-related aberrant cytoplasmic SFPQ IRTs and concurrent SFPQ protein mislocalization.^5^ Here we tested the hypothesis that aberrant cytoplasmic intron retention is a generalizable transcriptomic phenomenon in ALS. We examined patterns of splicing using the RNAseq pipeline VAST-TOOLS.^19^ In line with our previous study, increased intron retention was the dominant feature of the splicing programme during early neural differentiation in both the nucleus and the cytoplasm (Supplementary Fig. 1B–E). We identified 791 nuclear (527 included and 264 skipped) and 329 cytoplasmic (204 included and 125 skipped) alternative splicing events that are statistically significantly different between VCP^*mu*^ and control samples in at least one time point (Supplementary Fig. 1F and G). In line with our previous study, the majority of inclusion events between VCP^*mu*^ and control samples were retained introns (Fig. 1F, top). We found that these events peak in pMNs (Day 14 *in vitro,* DIV = 14) (Fig. 1F, bottom) when we observe a coincident decrease in expression of splicing factors (Fig. 1G and Supplementary Fig. 1H); most notable are the 112 aberrant intron retention events in the cytoplasmic fraction. Given that most VCP-driven aberrant retained intron events peak at Day 14, we subsequently chose to focus on this time point in the following analysis. Collectively, these findings demonstrate that aberrant cytoplasmic intron retention is a widespread phenomenon in VCP mutation-related ALS that occurs at an early stage during motor neurogenesis.

### A nucleocytoplasmic taxonomy for aberrant IRTs

**Figure 2:**
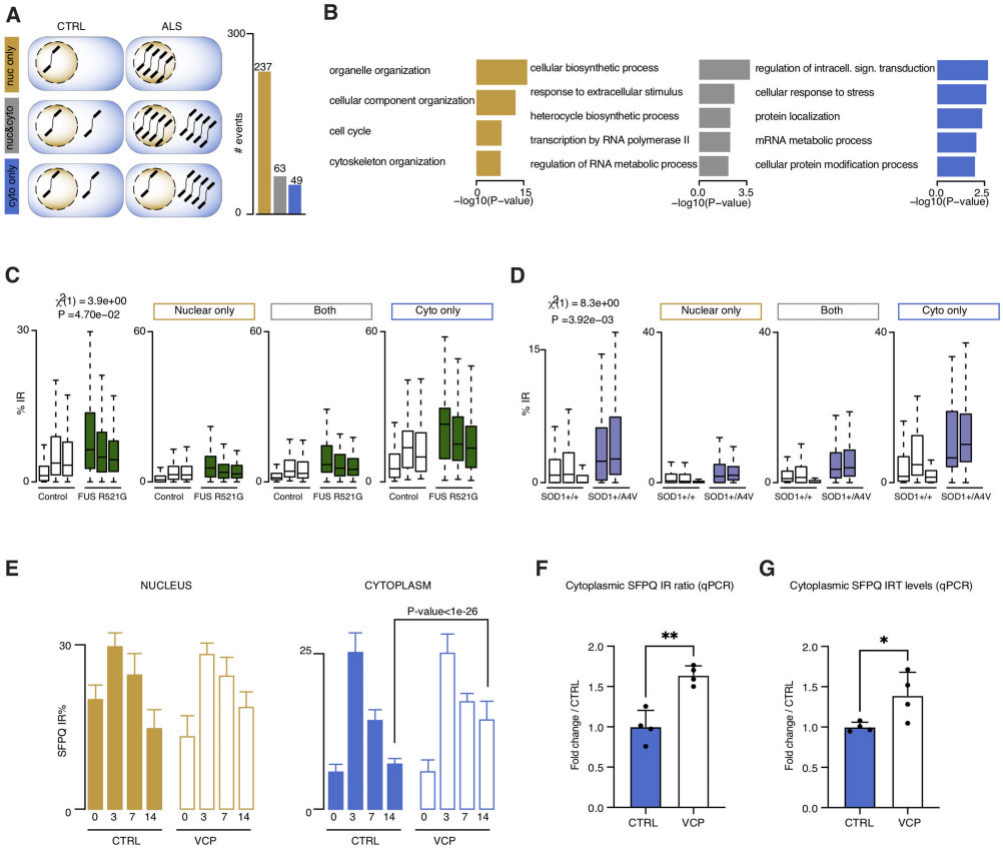
**Aberrant nuclear and cytoplasmic intronic sequences exhibit distinct characteristics.** (**A**) Schematic of our proposed taxonomy for aberrant IRTs (*left*) and bar graphs (*right*) representing the numbers of retained introns in VCP^*mu*^ compared to control samples at DIV = 14 that are predominantly nuclear (gold), in both the nucleus and cytoplasm (grey), or predominant in cytoplasm (blue). The number of events in each category is indicated above the bar. (**B**) Bar plots displaying the enrichment scores for GO biological functions of genes that are targeted by each group of aberrantly retained introns. (**C** and **D**) Box plots displaying the distribution of percentage retention for all 349 manually curated retained introns, 237 nuclear retained introns (gold), 63 cytoplasmic and nuclear retained introns, and 49 cytoplasmic retained introns in control motor neurons (white boxes), FUS mutant motor neurons (green boxes; **C**) or SOD1 mutant motor neurons samples (blue boxes; **D**).^20^^,^^21^ Mutant samples systematically exhibit a higher proportion of intron retention compared with controls. *P*-values obtained from linear mixed models accounting for idiosyncratic variations between the iPSC lines. Data shown as box plots in which the centre line is the median, limits are the interquartile range and whiskers are the minimum and maximum. (**E**) Bar graphs quantifying percentage intron retention in SFPQ transcripts at DIV = 0, 3, 7 and 14 in control and VCP*^mu^* samples (mean ± SD; Fisher count test) in the nucleus (*left*) and cytoplasm (*right*). (**F**) Bar graph showing intron retention levels analysed by qPCR at DIV =14 in control and VCP*^mu^* cytosolic fractions for SFPQ and measured by normalizing the levels of SFPQ IRT over the SFPQ expression level for each line. (**G**) Abundance of SFPQ IRT in the cytoplasm at DIV = 14 measured by qPCR and normalized over the compartment-specific housekeeping genes *NIT1* and *NFX1*. In **F** and **G**, data are expressed as fold-change over the control group mean; data displayed as bar plots with mean ± SD from four lines per group, with each data-point representing the average across two technical replicates (*P < 0.05, **P < 0.01, unpaired *t*-test).

We next manually curated the list of nuclear and cytoplasmic VCP mutation-related aberrant intron retention events, focusing on pMNs (DIV = 14). We identified three categories of aberrant IRTs in VCP^*mu*^cultures: (i) 237 predominantly in the nucleus; (ii) 63 in both the nucleus and the cytoplasm; and (iii) 49 predominantly in the cytoplasm (Fig. 2A and Supplementary Tables 3–5). Gene ontology functional enrichment analysis showed the specific biological association of affected transcripts, including cell cycle for the predominantly nuclear IRTs and protein localization for those that are predominantly cytoplasmic (Fig. 2B). To address whether these IRTs exist in motor neurons carrying other ALS causing mutations, including in FUS and SOD1, we probed external whole-cell RNAseq datasets and confirmed the presence of aberrant IRTs in these two genetically diverse forms of ALS. Notably the SOD1 data are derived from isogenic pairs and further allow us to confirm that the IRTs observed are mutation-dependent. We found that although our high-confidence set of 349 aberrant IRTs are also generally affected in FUS and SOD1 mutant motor neurons, the pool of 49 IRTs that is predominantly affected in the cytoplasm of VCP mutants exhibits the strongest effect in both FUS and SOD1 mutant motor neurons (Fig. 2C and D). Next, looking at the cytoplasmic abundance of the IRTs relative to the spliced transcripts, we found that the IRTs account for ∼40% of the absolute amount of transcript in the cytoplasm of VCP mutant cultures compared to ∼20% in the cytoplasm of control samples. This result indicates that the cytoplasmic IRTs abound in our iPSC model and are significantly increased by ALS-causing VCP mutations (Supplementary Fig. 2A). We previously identified aberrant intron retention in SFPQ in VCP^*mu*^ cultures at an early stage of motor neuron development,^5^ which we validated here again by RNAseq and RT-PCR (Fig. 2E and F and Supplementary Fig. 2B). Importantly, we validated three further IRTs (*OGT*, *TUSC3* and *DDX39*) using RT-PCR, where we not only confirmed the increase in intron retention ratio (Supplementary Fig. 2C), but also demonstrated an increase in cytoplasmic abundance of these IRTs (Fig. 2G and Supplementary Fig. 2D–E). This finding was present when normalizing using either cell compartment specific or whole cell housekeeping genes (Fig. 2G and Supplementary Fig. 2C–E, respectively). The finding that a large number of IRTs, including *SFPQ*, *OGT*, *TUSC3* and *DDX39*, exhibit a specific increase in cytoplasmic abundance in VCP*^mu^* cultures suggests that VCP mutations lead to aberrant nuclear export and/or cytoplasmic stabilization of a specific class of IRTs, potentially underlying stereotyped cellular dysfunction as a consequence.

### Aberrant predominantly cytoplasmic IRTs abundantly bind RBPs

**Figure 3:**
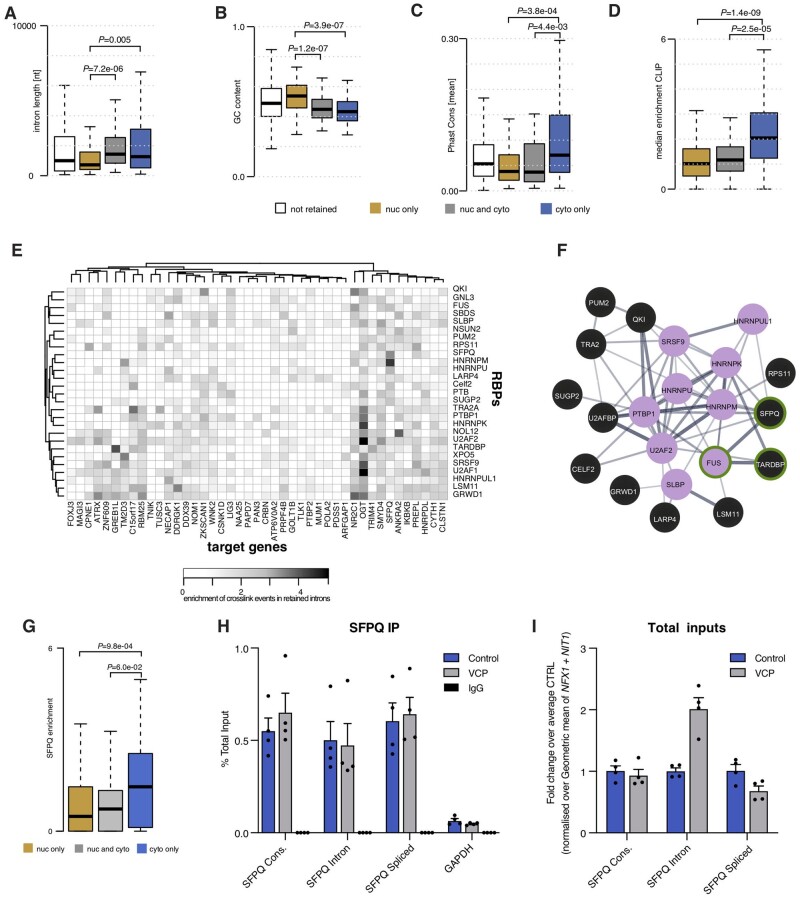
**Cytoplasmic IRTs create a mislocalization-prone environment for bound RBPs.** (**A**–**D**) Comparison of intron length, GC content (%), conservation scores and median enrichment for RBP binding sites of the three groups of aberrantly retained introns. Box plots are as shown in Fig. 1. (**C** and **D**) *P*-values obtained from Mann-Whitney test. All introns in the gene-set targeted by intron retention in VCP^*mu*^ at DIV = 14 (white). (**E**) Heat map of the enrichment score of the crosslinking events in each of the 49 predominantly cytoplasmic aberrant IRTs for 27 RBPs that exhibit significantly higher enrichment compared to the two other categories of IRTs (i.e. predominantly nuclear and those that are both cytoplasmic and nuclear). (**F**) Network of protein–protein interactions for 21 (out of the 27) RBPs for which binding sites are enriched in cytoplasmic aberrant retained introns. Edges represent experimentally determined protein–protein interactions annotated in the STRING database.^24^ Nine of these RBPs belong to the ‘Processing of Capped Intron-Containing Pre-mRNA’ Reactome^25^ pathway (filled magenta circles) and three are RBPs that exhibit hallmark nuclear-to-cytoplasmic mislocalization ALS (green circle). Line thickness indicates the strength of data support based on text mining and experiments. (**G**) Comparison of enrichment across all genes within each category for SFPQ crosslinking events in the retained introns between the three groups of aberrantly retained introns. (**H**) RNA immunoprecipitation (IP) performed on the cytoplasmic lysates from control and VCP^*mu*^ at DIV = 14 using antibodies for SFPQ or normal IgG as negative control. Levels of associated mRNA transcripts were analysed by qRT-PCR using primers designed against the indicated targets (*n* = 4). See also Supplementary Fig. 3A. (**I**) Bar graphs showing qRT-PCR analysis of levels of indicated transcripts in total input of cytoplasmic lysates from DIV = 14 control and VCP^*mu*^ samples. Values were normalized to the geometric mean of two compartment-specific housekeeping genes, *NFX1* and *NIT1*, before being expressed as fold-change over control group mean. *P < 0.05 Mann-Whitney test, *n *=* *4 lines per group.

Prior studies have shown that retained introns are on average shorter and more G/C rich.^2^^,^^22^^,^^23^ Strikingly, here we found that only the predominantly nuclear aberrantly retained introns exhibit these features. In complete contrast, aberrantly retained introns within the cytoplasm (including both those present within the nucleus and cytoplasm, and those in the cytoplasm predominantly) are on average longer and have lower GC content (Fig. 3A and B). Furthermore, the predominantly nuclear aberrant IRTs correlate with a cytoplasmic decrease in gene expression of their non-intron-retaining counterparts: this is consistent with prior observations showing that nuclearly detained IRTs reduce the level of gene expression.^2^ Conversely, IRTs found in the cytoplasm correlate with increased gene expression within the nucleus (Supplementary Fig. 2F). Importantly, this suggests that previously reported features^2^^,^^22^^,^^23^ discriminate nuclearly detained IRTs from cytoplasmic ones. Two additional features further discriminate cytoplasmic-predominant events from those found in both compartments: (i) a high conservation score (Fig. 3C); and (ii) a greater abundance of RBP crosslinking events in the predominantly cytoplasmic retained introns^26^^,^^27^ (Fig. 3D).

### Aberrant cytoplasmic IRTs are a blueprint for RBP mislocalization in VCP-related ALS

The finding that cytoplasmic aberrant IRTs abundantly bind to RBPs raises the hypothesis that this interaction drives hallmark RBP mislocalization events in ALS. Indeed, we previously reported that the *SFPQ* IRT and the SFPQ protein itself, which are predicted to avidly bind to each other, are exported to the cytoplasm thus providing a potential mechanism for SFPQ protein mislocalization in ALS.^5^ We therefore further examined the nature of the interaction between RBPs and aberrant cytoplasmic IRTs using our richer dataset. At least 27 RBPs systematically exhibit statistically significant increased binding to cytoplasmic-predominant retained introns compared with their nuclear-predominant counterparts (Fig. 3E). These RBPs form a densely connected network of experimentally validated interacting proteins that are enriched in mRNA metabolism functions (Fig. 3F). The network includes a subset of nine RBPs with known functions in processing capped intron-containing pre-mRNA, which further implicates disrupted post-transcriptional splicing in ALS pathogenesis. Importantly, also within this network of RBPs are those that exhibit hallmark nuclear-to-cytoplasmic mislocalization in ALS: SFPQ (Fig. 3G), transactivation response DNA binding protein 43 (TDP-43), and FUS (Supplementary Fig. 2H).^5^^,^^14^^,^^15^

Notably, we have previously demonstrated reduced nuclear-to-cytoplasmic ratio of SFPQ, FUS and TDP-43 proteins in VCP mutant hiPSC-derived neural precursors and/or motor neurons.^5^^,^^15^^,^^18^^,^^28^^,^^29^ To contextualize these previous protein mislocalization findings with our current study, we next sought to examine the mechanistic relationship between RBP function and aberrant accumulation of IRTs in the cytoplasm, focusing on the interaction between SFPQ protein and *SFPQ* IRTs. We first performed RNA immunoprecipitation to demonstrate that the SFPQ protein physically interacts with *SFPQ* IRT (Fig. 3H and Supplementary Fig. 3A). When considered together with the fact that we also found a higher abundance of *SFPQ* IRT in the input from the VCP mutant when compared to input from the control or the spliced/constitutive transcripts in either genotype (Fig. 3I), these data are consistent with an increased specific interaction between *SFPQ* IRT and SFPQ protein in VCP mutant cultures compared to control counterparts.

**Figure 4:**
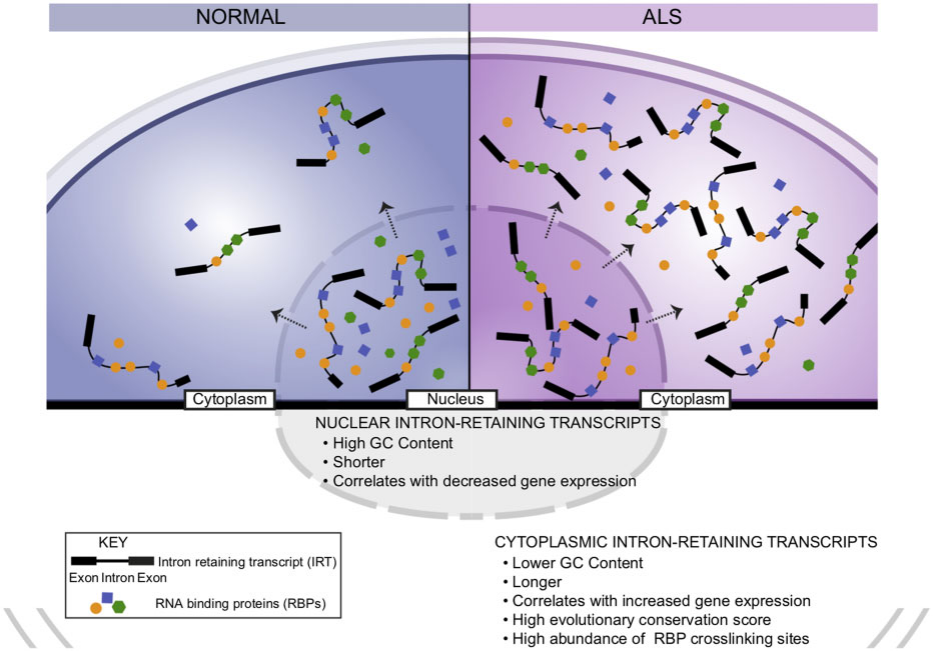
**Schematic of proposed model**‘ **where cytoplasmic IRT accumulation in ALS leads to protein mislocalization.**

We next knocked down SFPQ protein using an siRNA approach (Supplementary Fig. 3B and C). We found that while this siRNA-mediated knockdown resulted in a substantial reduction in expression of both *SFPQ* spliced transcript and *SFPQ* IRT (Supplementary Fig. 3B, D and E), a significant increase in the *SFPQ* intron retention ratio relative to the spliced transcript was observed (Supplementary Fig. 3F). This result suggests that SFPQ protein autoregulates its own intron retention, which is consistent with a recent study reporting significant retention of long introns upon SFPQ depletion.^30^^,^^31^ Altogether our data lend support to a model whereby ALS leads to an increase in the abundance of a class of cytoplasmic IRTs with a large capacity for binding RBPs. In turn, this may create an environment that encourages nuclear-to-cytoplasmic mislocalization of IRT-bound RBPs followed by nuclear loss of function and/or altered function in the cytoplasm (Fig. 4).

## Discussion

Recent studies have demonstrated that intron retention is more frequent in mammals than previously recognized.^1–4^ We previously identified aberrant cytoplasmic intron retention in the *SFPQ* transcript across human stem cell models of diverse genetic forms of ALS (including those caused by mutations in *VCP*, *SOD1* and *FUS* genes).^5^ In the present study, we sought to understand the generalizability of cytoplasmically localized aberrant IRTs in ALS by combining directed differentiation of patient-specific hiPSCs into spinal motor neurons with cellular fractionation and deep [poly(A)] RNA sequencing. We showed that aberrant cytoplasmic intron retention is indeed a widespread molecular phenomenon in ALS that comprises at least 112 transcripts including *SFPQ*. Furthermore, we specifically demonstrated an increase in the cytoplasmic abundance of the intronic sequences, suggesting aberrant nuclear export and/or cytoplasmic stabilization of a specific pool of IRTs in VCP mutant samples. Importantly, a significant overlap of these aberrant events has been previously recapitulated in RNAseq datasets from terminally differentiated motor neurons carrying mutations in *SOD1* or *FUS* genes.^20^^,^^21^ Furthermore, the most significant intron retention event identified in our aforementioned study, intron 9 of the *SFPQ* transcript, has been recently reproduced in human post-mortem tissue from sporadic ALS cases, demonstrating the predictive power of our hiPSC model.^32^

To better understand the nature of cytoplasmic IRTs, we categorized the aberrant intron retention events into three classes according to their nucleocytoplasmic distribution (predominantly nuclear, predominantly cytoplasmic and those present in both compartments). We chose to direct our attention to Day 14 of our motor neurogenesis protocol as this was where the majority of aberrant intron retention events were observed. Retained introns during neurogenesis have previously been shown to exhibit a highly correlated set of *cis* features comprising an ‘intron retention code’ that can reliably discriminate retained from constitutively spliced introns.^2^ Examining the *cis* attributes of the three categories of IRTs, we found that aberrantly retained introns that are predominantly in the nucleus of VCP mutant cultures display similar lengths and GC content to this ‘intron retention code’.^2^ Intron retention has been previously implicated in fine-tuning the cellular transcriptome by targeting transcripts to RNA degradation pathways such as nonsense-mediated decay.^1^^,^^2^^,^^7–9^ The retained introns characterized in the aforementioned studies act broadly to reduce the levels of transcripts that are not required.^3^^,^^33^ Indeed, the category of predominantly nuclear aberrant IRTs also correlates with reduced gene expression in our data. Remarkably, however, the two other categories we identified exhibit an almost opposite effect on their gene expression levels and thus stimulated further investigation. Our study further revealed a specific class of cytoplasmic IRTs that (i) have unique features compared to those reported in previous studies; and (ii) have conspicuously high affinity for RBPs, including those that are mislocalized in ALS (TDP43, FUS and SFPQ).^5^^,^^14^^,^^15^ These findings raise the hypothesis that a subset of cytoplasmic IRTs has a distinct role compared to the previously reported IRTs that regulate gene expression and translation through coupling with nonsense-mediated decay.^2^

RNA localization to distinct subcellular compartments has been shown to regulate spatio-temporal control of protein expression^34^ but less is known about their role in protein localization. Here we demonstrate for the first time a direct interaction between SFPQ protein and *SFPQ* IRT in the cytoplasm and further show siRNA SFPQ knockdown-related increase in *SFPQ* intron retention ratio relative to the spliced transcript. These data support a model where nuclear SFPQ binding to its retained intron facilitates splicing under normal circumstances, which is consistent with a recent study showing significant retention of long introns upon SFPQ depletion.^31^ An increase in *SFPQ* IRT cytoplasmic abundance may then lead to SFPQ nuclear-to-cytoplasmic mislocalization, SFPQ nuclear loss-of-function and consequently amplify aberrant splicing of *SFPQ* intron 9, which in turn exacerbates SFPQ nuclear loss-of-function. However, we have not formally excluded the possibility that an increase in *SFPQ* IRT upon knockdown is caused by differential accessibility or targeting of the *SFPQ* IRTs by the siRNAs. Therefore, future experiments should further address the specific role of nuclear SFPQ protein in regulating retention of intron 9 within its own transcript. Altogether we propose that a subset of IRTs aberrantly accumulate in the cytoplasm and their intronic sequences serve as ‘blueprints’ for the hallmark protein mislocalization events in ALS by creating a mislocalization-prone environment for their bound (and otherwise predominantly nuclear) RBPs (Fig. 4). This is reinforced by the fact that the RBPs with the largest difference in binding affinity between the predominantly cytoplasmic versus predominantly nuclear aberrant intron retention are those known to be mislocalized in ALS: TDP-43, FUS and SFPQ. However, further work is needed to definitively resolve the nature of the interaction between cytoplasmically mislocalized RBPs and cytoplasmic IRTs.

The first systematic characterization of intron retention during neuronal lineage restriction examined time-resolved RNAseq data during the differentiation of cortical glutamatergic neurons from murine embryonic stem cells. This study established that intron retention progressively increases during neuronal differentiation and downregulates non-physiologically relevant transcripts.^2^ Drawing on RNAseq data from our established hiPSC model,^18^ we subsequently reported a transient developmental intron retention programme early during neurogenesis from human pluripotent stem cells.^5^ It is noteworthy that the majority of studies have focused on nuclear IRTs and that the importance of cytoplasmic IRTs remains relatively understudied, particularly in the contexts of neuronal development and disease. In the present study we show that a large proportion of the transcripts exhibiting transient intron retention during neuronal development are indeed not restricted to the nucleus but transiently localize to the cytoplasm, and that this pool has strong binding affinity for RBPs. Based on these findings we hypothesize that a subset of neurodevelopmentally-regulated IRTs is specifically targeted to the cytoplasm where they attract RBPs through direct interaction leading to a transient decrease in splicing machinery in the nucleus. This adds a new and interesting complexity to the potential roles of IRTs, including in regulating gene expression during neuronal development.^2^ Future studies will directly assess this hypothesis. In summary, we propose that cytoplasmic-retained introns function as RNA regulators in the homeostatic control of RBP localization and that an ALS-related aberrant increase in cytoplasmic intron retaining transcripts disrupts this process, resulting in the hallmark RBP mislocalization phenotypes.

## Supplementary Material

awab078_Supplementary_Data

## Abbreviations

ALS: amyotrophic lateral sclerosis
hiPSC: human induced pluripotent stem cell
IRT: intron-retaining transcript
RBP: RNA binding protein
SFPQ: splicing factor proline and glutamine rich
