# Supplementary material for: Aberrant cytoplasmic intron retention is a blueprint for RNA binding protein mislocalization in VCP-related amyotrophic lateral sclerosis
Source: Brain. 2021 Mar 9;144(7):1985–93. doi: 10.1093/brain/awab078 (PMC8370440; doi:10.1093/brain/awab078)
Supplement: awab078_Supplementary_Data [file awab078_supplementary_data.zip › Supplementary Material.pdf]

SUPPLEMENTARY MATERIAL

An aberrant cytoplasmic intron retention programme is a blueprint for RBP mislocalization in VCP-related ALS

Giulia E. Tyzack[1,2,], Jacob Neeves[1,2,*], Hamish Crerar[1,2], Pierre Klein[1,2], Oliver Ziff[1,2], Doaa M. Taha[1,2,3], Raphaëlle Luisier[4,#], Nicholas M Luscombe[1,5,6#], Rickie Patani[1,2,#]*

[1]The Francis Crick Institute, 1 Midland Road, London NW1 1AT, UK; [2]Department of Neuromuscular Diseases, UCL Institute of Neurology, Queen Square, London, UK;
[3] Zoology Department, Faculty of Science, Alexandria University, Alexandria 21511, Egypt
[4]Idiap Research Institute, Martigny, Switzerland; [5]UCL Genetics Institute, University College London, Gower Street, London WC1E 6BT, UK; [6]Okinawa Institute of Science & Technology Graduate University, Okinawa 904-0495, Japan;
**These authors contributed equally to this work.*
[#]These authors contributed equally to this work.

SUPPLEMENTARY FIGURES AND TABLES

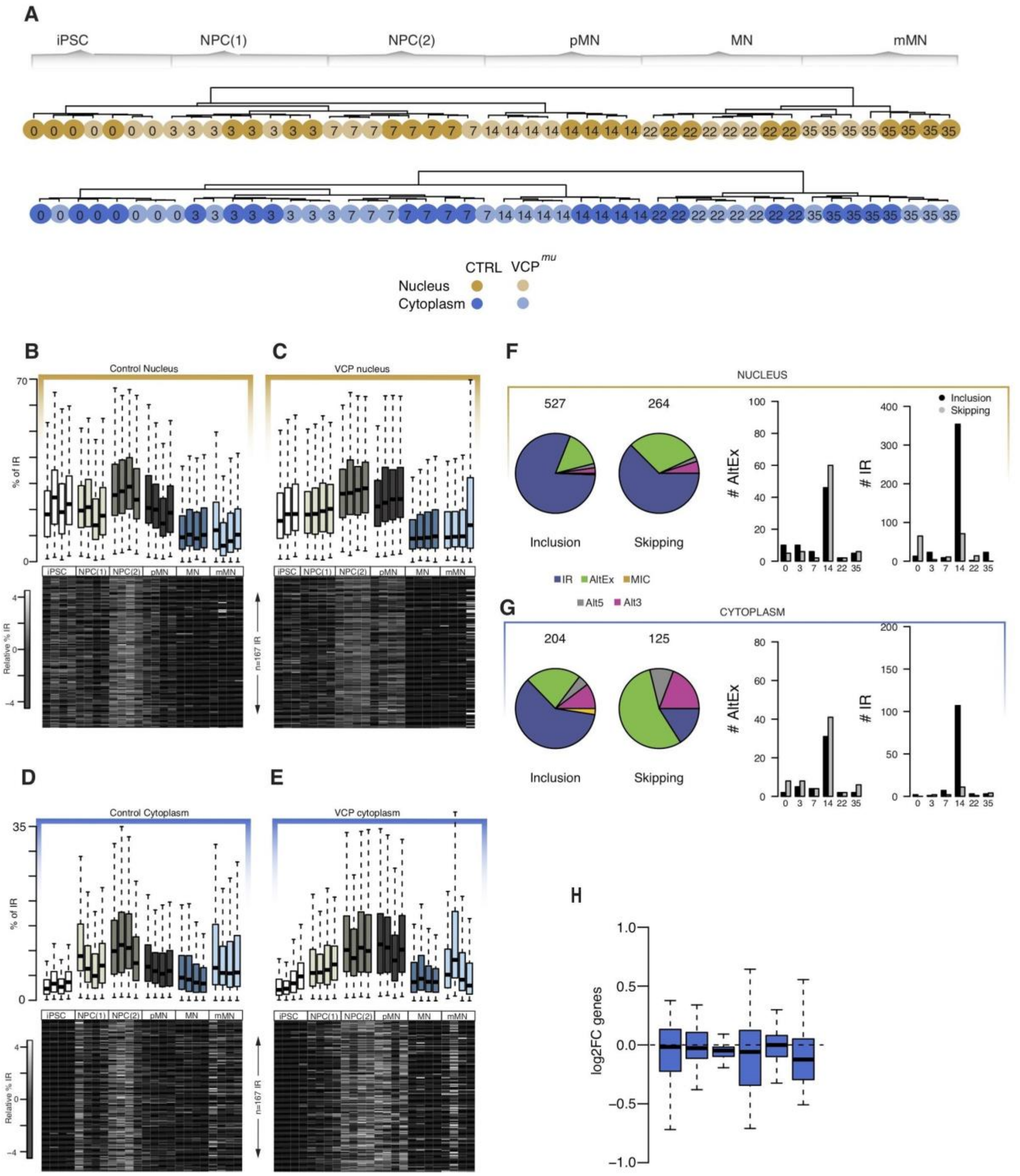

Supplementary Figure 1 | A. Unsupervised hierarchical clustering of 18,834 genes groups the 47 nuclear samples (upper) and 48 cytoplasmic samples (lower) according to developmental stage, rather than genetic background. Dark gold circles = nuclear control samples; light gold circles = nuclear VCP^{mu} samples; Dark blue circles =cytoplasmic control samples; light blue circles =cytoplasmic VCP^{mu} samples; sampling time-points are indicated

inside the circles. **B, C, D, E.** Heatmaps of the standardised relative percentage of IR in 167 introns identified in (Luisier *et al.*, 2018) in replicate samples at each differentiation stage in nuclear control samples, nuclear VCP^{mu} samples, cytoplasmic control samples, cytoplasmic VCP^{mu} samples respectively. **F, G.** (left) Pie charts representing proportions of all included and skipped splicing events in VCP^{mu} at any stages of motor neurogenesis compared with age-matched control samples in nuclear and cytoplasmic fractions respectively. Total numbers of events are indicated above the chart. Intron retention (IR); alternative exon (AltEx); microexons (MIC); alternative 5′ and 3′ UTR (Alt5 and Alt3). (right) Bar graphs representing the numbers of exonic (black bars) and intronic (grey bars) splicing events, in VCP^{mu} compared to control samples at specific timepoints during MN differentiation. **H.** Boxplots showing the distributions of nuclear log2 fold-changes for 72 essential splicing factor genes (**Table S8**) between VCP^{mu} and controls. Data shown as box plots in which the centre line is the median, limits are the interquartile range and whiskers are the minimum and maximum.

Supplementary Figure 2

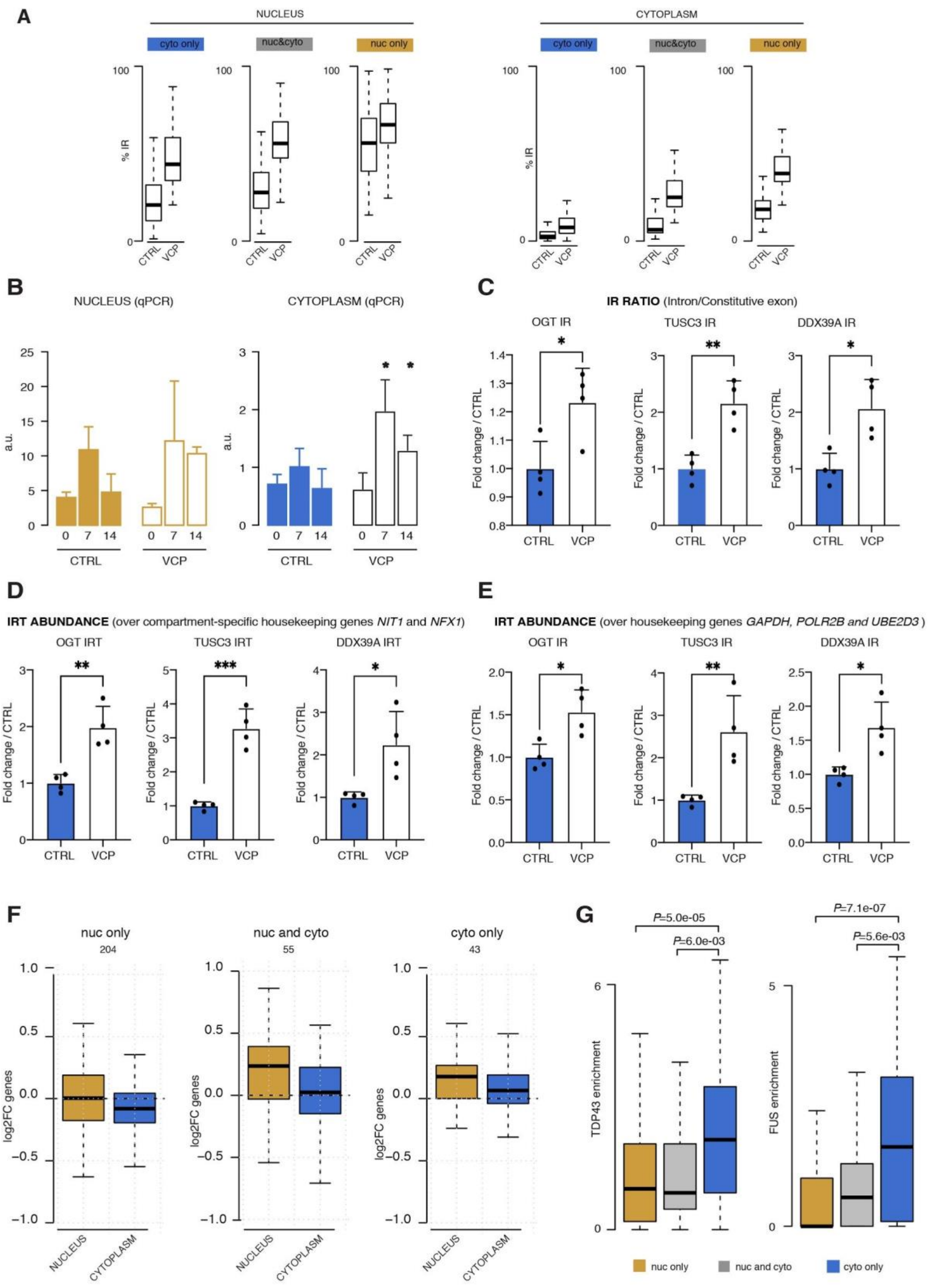

Supplementary Figure 2 | A. Comparison of the percentage of intron retention between control and *VCP*^{mu} samples in the nucleus and cytoplasm for intron retaining transcripts that are i) predominantly nuclear, ii) in both compartments, and iii) predominantly in the cytoplasm. **B.** Bar plots displaying SFPQ IR levels measured in the nucleus (left) and cytoplasm (right) by qPCR at DIV=0, 7, 14 in control and *VCP*^{mu} samples. SFPQ IR levels were measured normalising the levels of SFPQ IRT over the SFPQ expression level for each line. (mean±s.d. from four lines per group, *p<0.05, individual t-tests comparing control and *VCP*^{mu} samples at each time point). **C.** Bar plots showing IR levels measured by qPCR at DIV =14 in control and *VCP*^{mu} cytosolic fractions for OGT, TUSC3 and DDX39A transcripts. Proportion of intron retention was calculated as in (**B**). **D, E.** Abundance of OGT, TUSC3 and DDX39A IRTs in the cytoplasmic lysates at DIV=14 normalised over either the geometric mean of the compartment specific housekeeping genes NIT1 and NFX1 (**D**) or over the housekeeping genes GAPDH, POLR2B and UBE2D3 (Luisier *et al.*, 2018)**. E.** In C-E data is expressed as fold change over the control group mean; data displayed as bar plots with mean±s.d. from four lines per group, with each datapoint representing the average across two technical replicates (*p<0.05, **p<0.01, ***p<0.001, unpaired t-tests or non-parametric Mann Whitney tests comparing control and VCP^{mu} samples, following Shapiro Wilks test of normality, for each graph)**. F.** Boxplots showing the distributions of nuclear (gold) and cytoplasmic (blue) log2 fold-changes for 204, 55 and 43 genes belonging to the three groups of aberrant IR between VCP^{mu} and controls. Data shown as box plots in which the centre line is the median, limits are the interquartile range and whiskers are the minimum and maximum. **G.** Comparison of enrichment for TDP-43 and FUS crosslinking events between the 3 groups of aberrantly retained introns. Predominantly nuclear (gold), nuclear and cytoplasmic (grey), and predominantly cytoplasmic (blue). Data shown as box plots in which the centre line is the median, limits are the interquartile range and whiskers are the minimum and maximum. P-values obtained from Mann-Whitney test.

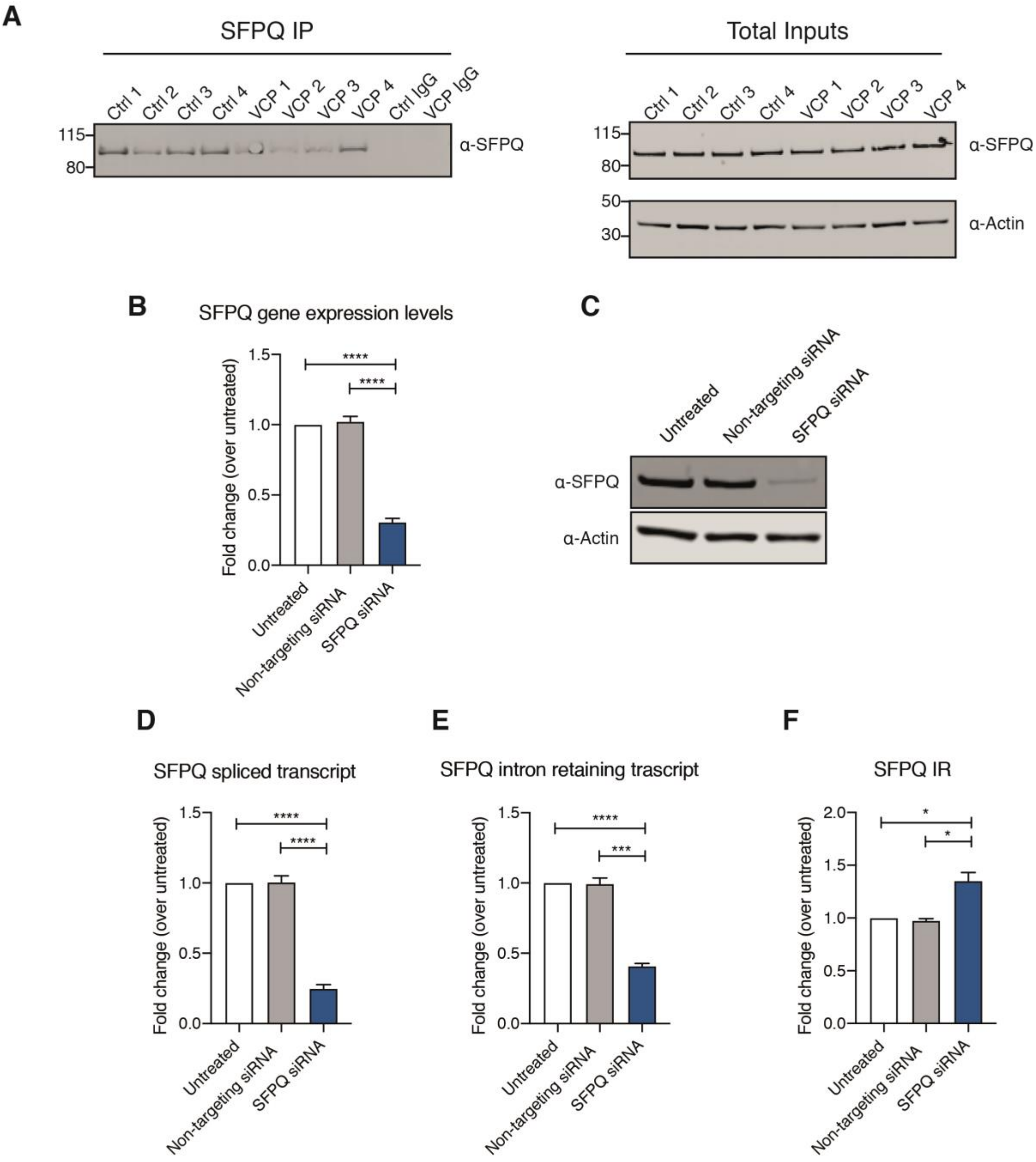

Supplementary Figure 3. A. Western blot analysis of SFPQ immunoprecipitation performed on cytoplasmic lysates from DIV=14 control or VCP^{mu} samples. Lysates from control or VCP^{mu} samples were pooled for IgG controls (left panel). 10% of lysate was run as total input (right panel). **B-F.** SFPQ Knockdown in iPSC-derived motor neurons (MNs). Untreated cells, and cells transfected with non targeting siRNAs were used as negative controls. Efficiency of SFPQ knockdown was measured at transcript level by qPCR using a primer pair designed against constitutive exons (**B**) and at protein level by Western blot (**C**). **D.** Levels of spliced SFPQ measured using primers designed on the two exons flanking intron 9. **E.** Levels of SFPQ

intron retaining transcript (IRT) using primers designed on intron 9. In B, D, E the levels of each transcript were normalised using GAPDH as housekeeping gene. **F.** Proportion of intron retention measured by normalising the levels of SFPQ IRT over the SFPQ expression level for each line. Data is expressed as average ± SEM from N=5 control lines, N=2 experimental blocks, analysed in technical duplicate. *p<0.05, ***p<0.001, ****p<0.0001. One-way ANOVA with Tukey correction for multiple comparisons.

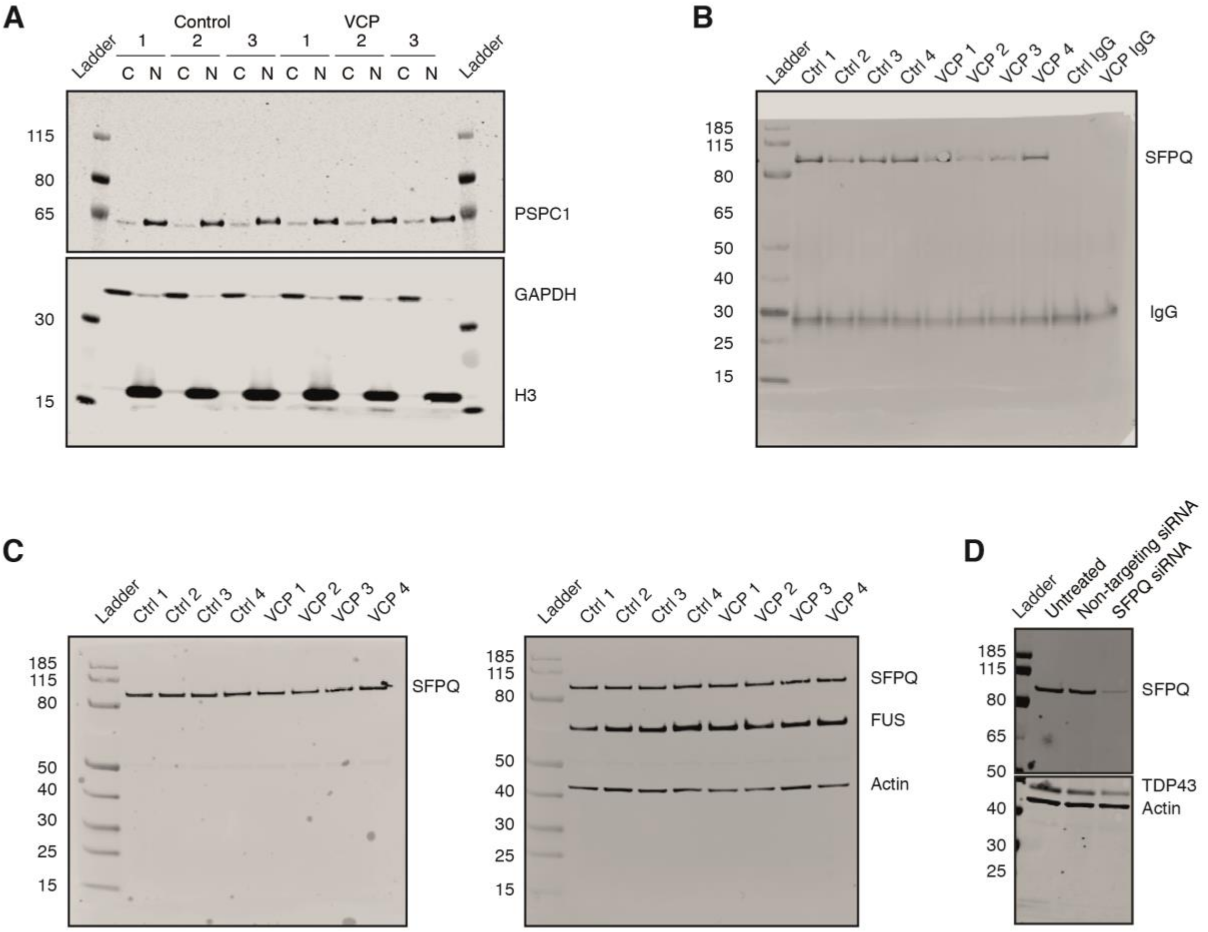

Supplementary Figure 4. Full length blots for figure 1B (**A**), Supplementary Figure 3A (**B**: SFPQ IP; **C**: Total inputs) and Supplementary figure 3C (**D**). The PageRuler Plus Prestained Protein Ladder was used as molecular weight marker in all blots. Samples were run on 4-12% Bis-Tris gels using MOPS running buffer. Membranes in A and D were cut at 50kDa using the protein ladder as reference to allow for multiple detection of different protein targets.

SUPPLEMENTARY TABLES 1-9 can be accessed here.

Table S1 | Description of the iPSC lines and RNA sequencing samples used in this study.

Table S2 | Detailed results of the quality control of the 288 fastq files.

Table S3 | List of the 237 IR events that are aberrant at DIV=14 in VCP *nuclear* compartment predominantly.

Table S4 | List of the 63 IR events that are aberrant at DIV=14 in VCP *nuclear* and *cytoplasmic* compartments.

Table S5 | List of the 49 IR events that are aberrant at DIV=14 in VCP *cytoplasmic* compartment predominantly.

Table S6 | Introns characteristics in terms of length, GC content, maximum entropy at their extremity and conservation scores.

Table S7 | List of primers for qPCR validation of aberrant intron retention.

Table S8 | List of splicing factors used for Figure 1.

Table S9 | List of Antibodies used for assessment of nucleocytoplasmic fractionation efficiency and RNA-immunoprecipitation.

SUPPLEMENTARY METHODS

Motor neuron differentiation

Motor neuron (MN) differentiation was carried out using an adapted version of a previously published protocol (Hall *et al.*, 2017). Briefly, iPSCs were first differentiated to neuroepithelium by plating on Geltrex-coated plates to 100% confluency in chemically defined medium consisting of DMEM/F12 Glutamax, Neurobasal, L-Glutamine, N2 supplement, non essential amino acids, B27 supplement, β-mercaptoethanol (all from Life Technologies) and insulin (Sigma). Treatment with small molecules from day 0-7 was as follows: 1 µM Dorsomorphin (Millipore), 2 µM SB431542 (Tocris Bioscience), and 3.3 µM CHIR99021 (Miltenyi Biotec). Starting from day 8, the neuroepithelial layer was patterned for 7 days with 0.5 µM retinoic acid and 1 µM Purmorphamine. At day 14 pMN precursors were treated with 0.1 µM Purmorphamine for a further 4 days before being terminally differentiated in 0.1 µM Compound E (Enzo Life Sciences) to promote cell cycle exit. At relevant time points cells were harvested for cellular fractionation.

Cell fractionation

Biochemical subcellular fractionation was achieved for all cell stages using the Ambion PARIS kit (ThermoFisher Scientific) cell fractionation buffer, following the manufacturer's general protocol, and an 8 M Urea Nuclear Lysis Buffer prepared in house. Initially, cells were washed once using ice-cold PBS. Cytosolic fraction was then obtained by lysing cells directly in ice-cold cell fractionation buffer (ThermoFisher Scientific) for 3-15 minutes, thus disrupting plasma membranes, whilst leaving nuclear membranes intact. Lysates were then centrifuged for 3 minutes at 500 x *g* at 4°C. The supernatant was then collected, further centrifuged at maximum speed in a bench centrifuge at 4°C for 1 minute, and the resulting supernatant was then processed as cytosolic fraction. Nuclear pellets from the first centrifugation step were gently washed once with cell fractionation buffer and then lysed on ice for 30 minutes in 8 M Urea Nuclear Lysis Buffer, containing 50 mM Tris-HCL (pH 8), 100 mM NaCl, 0.1% SDS, and 1 mM DTT. The resulting nuclear fraction was then homogenised using a QIAshredder (QIAGEN) to shred chromatin and reduce viscosity, before being further processed for RNA extraction. Both lysis buffers were supplemented with 0.1 U/µl RiboLock RNase Inhibitor (ThermoFisher Scientific) and HALT Protease Inhibitor Complex (ThermoFisher Scientific).

RNA extraction and sequencing

The Promega Maxwell RSC simplyRNA cells kit including DNase treatment, alongside the Maxwell RSC instrument, was used for RNA extractions. The nanodrop was used to assess RNA concentration and the 260/280 ratio, and the Agilent TapeStationwas used to assess quality. RNA integrity (RIN) scores were analysed to quality check all samples used in this work.

RNA-sequencing data

Paired-end polyA stranded RNAseq libraries were prepared from fractionated nucleus and cytoplasm obtained from 6 distinct stages of motor neuron differentiation from control and VCP^{mu} samples (iPSC, and days 3, 7, 14, 21 and 35) using the NEBNext® Ultra™ II Directional RNA Library Prep Kit for Illumina®, with NEBNext® Poly(A) mRNA Magnetic Isolation Module with 500 ng of total RNA as input. Libraries were sequenced at the OIST DNA sequencing section using the NovaSeq 6000 Sequencing technology. 50 bp-long reads were trimmed for adapter sequence and initially aligned to ribosomal RNA sequences to filter out reads that may come from ribosomal RNA contamination using bowtie2 (-v 0) (Langmead and Salzberg, 2012). The remaining reads were aligned to the human genome (hg38) using the splice aware aligner STAR (STAR-2.6.0) (Dobin *et al.*, 2013) with default parameters. All libraries generated in this study had <1% rRNA, <1% mtDNA, >90% strandedness and >70% exonic reads (data not shown). One control iPSC nuclear sample failed quality control and was discarded. The list of samples together with the corresponding number of reads in each library and alignment statistics are provided in **Table S2**. All sequence data for this project has been deposited at NCBI GEO database under accession number **GSE152983**.

Gene expression analysis

Kallisto (Bray *et al.*, 2016) was used to (1) build a transcript index from the Gencode hg38 release Homo sapiens transcriptome (-k 31), (2) pseudo-align the RNA-seq reads to the transcriptome and (3) quantify transcript abundances (-b 100 -s 50—rf-stranded). Subsequent analysis was performed with the R statistical package version 3.3.1 (2016) and Bioconductor

libraries version 3.3 (R Core Team. R: A Language and Environment for Statistical Computing. Vienna, Austria: R Foundation for Statistical Computing; 2013). Kallisto outputs transcript abundance, and thus we calculated the abundance of genes by summing up the estimated raw count of the constituent isoforms to obtain a single value per gene. For a given sample, the histogram of log2 gene count is generally bimodal, with the modes corresponding to non-expressed and expressed genes. Reliably expressed genes for each condition (VCP^{mu} or control at days 0, 3, 7, 14, 22 and 35 in each fraction) were next identified by fitting a two-component Gaussian mixture to the log2 estimated count gene data with R package mclust (Fraley and Raftery, n.d.) ; a pseudocount of 1 was added before log2 transformation. A gene was considered to be reliably expressed in a given condition if the probability of it belonging to the non-expressed class was under 1% in each sample belonging to the condition. 18,834 genes were selected based on their detected expression in at least one of the 24 conditions (i.e. 6 different timepoints of lineage restriction for control and VCP^{mu} in nuclear and cytoplasm). Next we quantile normalized the columns of the gene count matrix with R package limma (Boldstad *et al.*, 2003). Unsupervised hierarchical clustering of the filtered and normalised gene count matrix was performed with Spearman rank correlation as a distance measure and complete clustering algorithm. Principal component analysis has been done with the svd function in R. Differential gene expression analysis was performed with Sleuth comparing VCP mutant or control at each day (0, 3, 7, 14, 22 and 35) in each fraction. Genes that showed a log twofold differential expression and a P-value < 0.05, and that were reliably expressed in either VCP mutant or control condition were considered as changing significantly.

Splicing analysis

Intron retention (IR) focussed analysis has been performed on the 167 IR events previously found to be retained during MN differentiation (Luisier *et al.*, 2018) for which a percentage of IR has been calculated as the fraction of intron mapping reads to the average number of reads mapping to the adjacent 5’ and 3’ exons normalised to the length of the respective intron and exons. A Fisher count test P-value has been obtained when testing for differential IR between conditions. Next the identification of all classes of alternative splicing (AS) events in motor neuron differentiation was performed with the RNA-seq pipeline *vast-tools* (Irimia *et al.*, 2014). For an AS event to be considered differentially regulated between two conditions, we required a minimum average ΔPSI (between the paired replicates) of at least 15% and that the

transcript targeted by the splicing event in question to be reliably expressed in all samples from the conditions compared i.e enough read coverage in all samples of interest. We next focussed on the introns aberrantly retained at day 14 in VCP mutant compared to control samples and conducted Integrative Genomics Viewer (IGV)-guided manual curation to remove low coverage IR obtaining 237 high-confidence IR events that were aberrantly retained predominantly in the nucleus, 63 introns aberrantly retained in both the nucleus and the cytoplasm, and 49 introns predominantly in the cytoplasm. These and their associated characteristics (GC content, conservation score, enrichment in CLIP binding sites) are reported in **Tables S3-S6**.

GO enrichment analysis

GO enrichment analysis was performed using classic Fisher test with topGO Bioconductor package (Alexa and Rahnenfuhrer, 2016). Only GO terms containing at least 10 annotated genes were considered. A P-value of 0.05 was used as the level of significance. On the figures, top significant GO terms were manually selected by removing redundant GO terms and terms which contain fewer than five significant genes.

Mapping and analysis of CLIP data

To identify RBPs that bind to aberrantly retained introns, we examined iCLIP data for 21 RBPs (Attig *et al.*, 2018), and eCLIP data from K562 and HepG2 cells for 112 RBPs available from ENCODE (Sloan *et al.*, 2016; Van Nostrand *et al.*, 2017). Before mapping the reads, adapter sequences were removed using Cutadapt v1.9.dev1 and reads shorter than 18 nucleotides were dropped from the analysis. Reads were mapped with STAR v2.4.0i (Dobin *et al.*, 2013) to UCSC hg19/GRCh37 genome assembly. The results were lifted to hg38 using liftOver (Hinrichs *et al.*, 2006) To quantify binding to individual loci, only uniquely mapping reads were used. Relative enrichment for each of the RBPs was obtained by computing the proportion of crosslink events mapping to retained intron compared to non-retained introns of the same genes.

Reverse transcription, qPCR and intron retention validation

Reverse transcription was performed using the Revert Aid First Strand cDNA Synthesis Kit (ThermoFisher Scientific) or Superscript IV (ThermoFisher Scientific) using 200ng-500ng of total RNA and random hexamers. qPCR was performed using the PowerUP SYBR Green Master Mix (ThermoFisher Scientific) and the Agilent Mx3000P QPCR System or the QuantStudio 6 Flex Real-Time PCR System (Applied Biosystems). Primers used are listed in **Table S7**. Specific amplification was determined by melt curve analysis and agarose gel electrophoresis of the PCR products. Primer pairs with 90-110% efficiency were used. Intron retention validation was performed as previously described (Luisier *et al.*, 2018), and RT-minus samples were used as negative controls. Data was analysed using the *ddCt* method and is expressed as fold change over control group.

Protein quantification and Western Blotting

Protein abundances were quantified using the DC assay (BIO-RAD). 10x NuPAGE reducing agent (ThermoFisher) and 4x NuPAGE LDS sample buffer (ThermoFisher) was added to approximately 10 ug protein sample and placed at 90°C for 10 minutes for denaturation. Samples were loaded onto an Invitrogen NuPAGE 4-12% Bis-Tris protein gel (ThermoFisher Scientific) and run at 180 V for 1-2 hours in NuPAGE MOPS running buffer (ThermoFisher Scientific). Protein was then transferred onto nitrocellulose membrane using semi-dry Transblot Turbo transfer system (BIO-RAD) or wet transfer (Invitrogen). Membranes were blocked for 1 hour in 5% milk in 0.1% PBS-T then incubated with primary antibodies (see **Table S9**) overnight at 4°C. After 3x 5 minute washes in PBS-T, IRdye 680 and 800CW secondary antibodies (1:10,000), in 5% milk solution, were added as appropriate for 1 hour at room temperature. Following a further 3 x 5 minute washes membranes were imaged using a LICOR FC Odyssey. Blots were quantified using ImageStudio Lite 5.2. Full length blots are shown in **Supplementary Figure 4**.

RNA immunoprecipitation

RNA immunoprecipitations were carried out as previously described (Crerar *et al.*, 2019) with the following modifications. Three μg SFPQ antibody (Abcam, ab11825), or Mouse IgG antibody (Santa Cruz sc2027) were incubated with prewashed protein G dynabeads (Thermo) in a PBS buffer containing 1 mg/ml heparin and 1% BSA for 2 hours 4°C, and then washed with wash buffer (50 mM Tris pH8, 150 mM NaCl, 1% Triton X-100). 100 μg of cytoplasmic

lysate from day 14 differentiated control or VCP^{mu} samples was incubated with antibody conjugated beads for 1 hour at 4°C in wash buffer containing 0.2 mg/ml heparin, 0.1 U/µl RiboLock RNase Inhibitor (Thermo) and Halt protease inhibitor cocktail. 2% of lysate volume was purified by the Maxwell RSC simplyCells kit (Promega) for total input. Beads were washed 6 times with wash buffer 5 minutes 4°C followed by elution of RNA in extraction buffer (0.2 M NaAcetate, 1 mM EDTA, 0.2% SDS) for 5 minutes at 70°C, or protein in 2x LDS 5% beta mercaptoethanol and boiled for 8 minutes at 95°C. RNA was purified from immunocomplexes using PureLink® RNA Micro Scale Kit according to manufacturer's instructions with on-column DNAse digestion, reverse transcribed with Superscript IV and random hexamers (Thermo) and then analyzed by qPCR.

siRNA-mediated knockdown in iPSC-derived motor neurons

Neural precursors were plated in 12-well plates in N2B27 media at a density of 2.5 x 10^5 cells/well and were transfected with 30pmol of siRNA directed against SFPQ (pool of 4 siRNAs, ON-TARGETplus SmartPool SFPQ siRNA, L-006455-00-0005, Horizon) or non-targeting siRNAs as negative control (pool of 4 siRNAs, ON-TARGETplus Non-targeting Control Pool, D-001810-10-05, Horizon). Lipofectamine RNAiMax (ThermoFisher Scientific) was used as transfecting reagent according to manufacturer's instructions. After overnight incubation, the media was changed to Compound E 0.1 uM in N2B27 to allow terminal differentiation to MNs (Hall *et al.*, 2017) and samples were harvested for either protein or RNA extraction 96 hours after transfection.

Contact for reagent and resource sharing

Further information and requests for resources and reagents should be directed to and will be fulfilled by the Lead Contact, Rickie Patani (rickie.patani@ucl.ac.uk).

REFERENCES FOR SUPPLEMENTARY MATERIAL

Alexa A, Rahnenfuhrer J. topGO: Enrichment Analysis for Gene Ontology. 2016

Attig J, Agostini F, Gooding C, Chakrabarti AM, Singh A, Haberman N, et al. Heteromeric RNP Assembly at LINEs Controls Lineage-Specific RNA Processing. Cell 2018; 174: 1067–81.e17.

Boldstad BM, Irizarry RA, Astrand M, Speed TP. A Comparison of Normalization Methods for High Density Oligonucleotide Array Data Based on Bias and Variance. Bioinformatics 2003; 19: 185–93.

Bray NL, Pimentel H, Melsted P, Pachter L. Near-optimal probabilistic RNA-seq quantification. Nat Biotechnol 2016; 34: 525–7.

Crerar H, Scott-Solomon E, Bodkin-Clarke C, Andreassi C, Hazbon M, Logie E, et al. Regulation of NGF Signaling by an Axonal Untranslated mRNA. Neuron 2019; 102: 553–63.e8.

Dobin A, Davis CA, Schlesinger F, Drenkow J, Zaleski C, Jha S, et al. STAR: ultrafast universal RNA-seq aligner. Bioinformatics 2013; 29: 15–21.

Fraley C, Raftery AE. mclust Version 4 for R: Normal Mixture Modeling for Model-Based Clustering, Classification, and Density Estimation.

Hall CE, Yao Z, Choi M, Tyzack GE, Serio A, Luisier R, et al. Progressive Motor Neuron Pathology and the Role of Astrocytes in a Human Stem Cell Model of VCP-Related ALS. Cell Rep 2017; 19: 1739–49.

Hinrichs AS, Karolchik D, Baertsch R, Barber GP, Bejerano G, Clawson H, et al. The UCSC Genome Browser Database: update 2006. Nucleic Acids Res 2006; 34: D590–8.

Irimia M, Weatheritt RJ, Ellis JD, Parikshak NN, Gonatopoulos-Pournatzis T, Babor M, et al. A highly conserved program of neuronal microexons is misregulated in autistic brains. Cell 2014; 159: 1511–23.

Langmead B, Salzberg SL. Fast gapped-read alignment with Bowtie 2. Nat Methods 2012; 9: 357–9.

Luisier R, Tyzack GE, Hall CE, Mitchell JS, Devine H, Taha DM, et al. Intron retention and nuclear loss of SFPQ are molecular hallmarks of ALS. Nat Commun 2018; 9: 2010.

Sloan CA, Chan ET, Davidson JM, Malladi VS, Strattan JS, Hitz BC, et al. ENCODE data at the ENCODE portal. Nucleic Acids Res 2016; 44: D726–32.

Van Nostrand EL, Freese P, Pratt GA, Wang X, Wei X. A large-scale binding and functional map of human RNA binding proteins [Internet]. bioRxiv 2017Available from: https://www.biorxiv.org/content/early/2017/08/23/179648.abstract
